# Supersymmetric Single-Lateral-Mode GaN-Based Ridge-Waveguide Edge-Emitting Lasers

**DOI:** 10.3390/ma18194453

**Published:** 2025-09-24

**Authors:** Łukasz Piskorski

**Affiliations:** Photonics Group, Institute of Physics, Lodz University of Technology, Wólczańska 217/221, 93-005 Łódź, Poland; lukasz.piskorski@p.lodz.pl

**Keywords:** semiconductor laser, edge-emitting laser, coupled ridges, computer simulation, nitride materials

## Abstract

High-power nitride-based edge-emitting lasers with low-divergence Gaussian beams are useful for applications including laser surgery, material processing, and 3D printing. Fundamental lateral mode operation is typically achieved using narrow or shallow ridges. However, narrow ridges limit the active region, while shallow ridges can allow higher-order mode lasing. To address these challenges, this study applies a supersymmetry approach using optical coupling between neighbouring ridges to confine the fundamental mode while suppressing higher-order modes. Two nitride-based edge-emitting laser configurations—double-ridge and triple-ridge waveguides—are analysed, with a focus on ridge-width tolerances and the effects of gain and absorption. Both configurations achieve strong mode discrimination. However, the triple-ridge waveguide structure exhibits a mode separation ratio more than twice that of the double-ridge waveguide, making it promising for high-power single-mode operation. The results of this study provide a basis for further study of supersymmetry effects in nitride lasers.

## 1. Introduction

High-optical-power nitride-based lasers delivering energy in a low-divergence Gaussian beam represent a competitive light source for a wide range of applications, including as pumping sources and in laser surgery [1], material processing [2], 3D printing [3], and many others [4]. Gaussian beams provide superior beam quality, low divergence, and high spatial coherence, making them well suited for highly accurate applications. In many cases, it is important to focus a laser beam down to the smallest possible spot, to maximize its intensity and minimize the affected area. Various sources of blue light are available, including Ti:Sapphire lasers [5], frequency-doubled Nd:YAG [6] lasers, and argon-ion lasers [7], but commercially available nitride-based diode lasers offer a particularly attractive solution. They combine compact size, high electrical-to-optical efficiency, low beam divergence, and high brightness, enabling precise control and having minimal thermal impact on surrounding areas. Their emission in the short-wavelength blue range allows tighter focusing and higher energy density, improving spatial resolution and reducing heat-affected zones compared to longer-wavelength sources. Additionally, their ability to generate low-divergence Gaussian beams and high beam quality makes them ideal for high-precision applications.

Fundamental lateral mode operation of edge-emitting lasers (EELs) is typically achieved by employing a ridge that is either sufficiently narrow or shallowly etched [8,9]. The ridge defines a waveguiding effect in the lateral direction as the effective refractive index within the ridge region is higher than that of the surrounding areas. A narrow ridge confines the fundamental transverse electric (TE) mode, which has no cut-off, while suppressing or cutting off higher-order TE modes. This design results in a limited area for current injection into the active layer with quantum wells (QWs). Consequently, the active region’s small volume restricts stimulated emission to limited area, leading to reduced optical power output. Furthermore, a narrow active area generates a high local thermal power density, making heat dissipation challenging.

In contrast, a shallow ridge introduces a small effective refractive index step in the lateral direction, allowing higher-order modes to be suppressed in broader ridges. This approach facilitates single-mode operation even with wider ridges, which could potentially enable higher optical power. However, the weak lateral waveguiding effect increases threshold current and the resulting heat generation introduces a refractive index distribution that strengthens the waveguiding effect, contributes to the confinement of higher-order modes under more intense excitation, and as a consequence leads to multiple lateral modes lasing [10,11,12].

To overcome these challenges and achieve fundamental lateral mode operation, this paper employs optical coupling between neighbouring laser ridges, also known as the supersymmetry (SUSY) approach [13]. SUSY has a long-standing history. It was initially proposed in the context of particle physics and later developed in quantum mechanics [14,15]. Due to the isomorphism between the Schrödinger and Helmholtz equations, it was also adopted by wave optics. This isomorphism further enabled the application of parity-time (PT) symmetry in wave optics [16], particularly in coupled waveguides [17] where optical coupling determines the modal characteristics of the emitted light.

A typical example of a SUSY manifestation is a system of two optically coupled different waveguides in which the propagation constants of all modes in both waveguides have the same values [18]. The exception is the fundamental mode in one of the waveguides, which does not have its mode ‘partner’ with the same propagation constant in the neighbouring waveguide. As a result, all modes which have partners will propagate in both waveguides simultaneously, with the exception of the fundamental mode which has no partner. This mode will only be localized in one of the waveguides.

**Figure 1:**
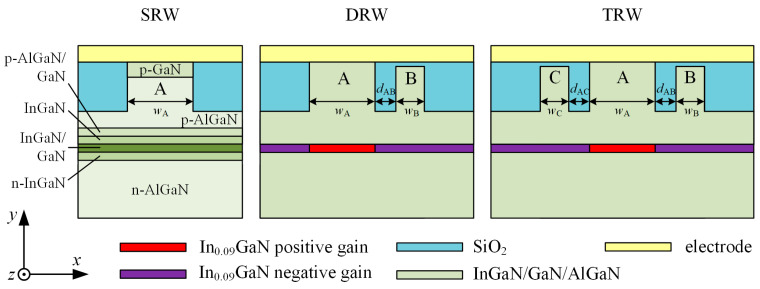
Schematic representation of the single-, double-, and triple-ridge waveguide edge-emitting laser structures used in the optical simulation, together with the coordinate system used in the analysis. Symbols: *w*_A_, *w*_B_, *w*_C_ denote the widths of ridges A, B, and C, respectively, while *d*_AB_, *d*_AC_ represent the spacings between ridges A and B, and ridges A and C, respectively.

In this paper, SUSY is applied to two configurations of nitride-based EELs (Figure 1) to enhance their fundamental-mode emission. In the first configuration, two optically coupled ridges are employed: a wider ridge, denoted as A, and a narrower adjacent ridge, denoted as B. This configuration is referred to as a double-ridge waveguide (DRW). In the second configuration, three optically coupled ridges are used, with the widest labelled A and the narrower ones labelled B and C. This arrangement is referred to as a triple-ridge waveguide (TRW). A conventional structure featuring a single ridge is referred to as a single-ridge waveguide (SRW). Throughout this article, the laser regions located directly beneath the ridges are referred to as apertures.

When the effective refractive indices of modes in two isolated ridges are equal, reducing the distance between the ridges to enable optical coupling results in field redistribution, with nonzero intensity present in both apertures. In the DRW configuration, if the widths of ridges A and B are chosen such that all higher-order ${\mathrm{TE}}^{DRW}$ modes in A have effective indices matched to modes in B, these modes couple and delocalize across both apertures. The fundamental mode, with no effective-refractive-index-matched counterpart in B, remains confined to ridge A. The same mechanism applies to the TRW configuration, where ridge widths are chosen so that higher-order ${\mathrm{TE}}^{TRW}$ modes in A match modes in B and C. In the DRW configuration, mode discrimination is limited to the first higher-order mode (${\mathrm{TE}}_{1}^{DRW}$), while the TRW configuration extends the suppression to both ${\mathrm{TE}}_{1}^{TRW}$ and ${\mathrm{TE}}_{2}^{TRW}$ higher-order modes. In this paper, the subscript used for the fundamental mode is 0. For higher-order modes, the subscript is a positive integer. As the focus is mainly on the discrimination of higher-order modes, the specific way in which these modes are labelled has no impact on the results. Therefore, the labelling can be chosen arbitrarily. Here, higher-order modes are enumerated in descending order of the real part of the effective refractive index.

The design principle of the SUSY laser is based on a straightforward condition: the ridge widths of B in the DRW configuration, and of B and C in the TRW configuration, are chosen such that the effective refractive index of each higher-order mode supported by ridge A matches that of the fundamental mode in ridge B (for DRW) or in ridges B and C (for TRW), when considered independently.

In this work, the tolerance of the ridge widths B and C is examined to ensure effective discrimination of higher-order modes in the DRW and TRW configurations. Additionally, the impact of variations in the imaginary part of the refractive index within the active region between A and B/C is investigated. The gain and absorption occurring in the apertures influence the imaginary component of the refractive index, potentially altering the combined effective refractive indices of the modes in A and in B/C. This variation can hinder optical coupling between apertures (PT symmetry), thereby promoting mode localization within individual apertures. This phenomenon is also known as PT symmetry breaking [19]. In this work, the ranges of gain and absorption values that ensure effective discrimination of higher-order modes are identified.

To the best of the author’s knowledge, this is the first simulation of its kind performed for nitride-based EELs. Although similar studies exist for devices based on arsenide [20,21,22] or phosphide materials [23], this work focuses on nitrides, which are characterized by smaller refractive index contrasts compared to arsenides and phosphides, and also by a shorter emission wavelength which also plays a significant role in the optical behaviour of the device.

## 2. Modelled Structures

The theoretical study presented in this paper is based on an experimentally realized EEL emitting at 430 nm [24], schematically shown in Figure 1. Its structure consists of an n-AlGaN cladding layer with a compositionally graded region at its top, an n-InGaN lower waveguide (WG) layer, two pairs of InGaN/GaN QWs, an upper i-InGaN WG layer, a compositionally graded p-AlGaN electron blocking layer (EBL), a p-AlGaN cladding layer with a graded doping profile and a compositionally graded region at its bottom, a p-GaN contact layer, and a metal contact on the top. A ridge waveguide (RW) structure is formed by replacing the side regions of the p-GaN contact and p-AlGaN cladding layers with SiO_2_, which is implemented by reactive ion etching and followed by plasma-enhanced chemical vapour deposition of SiO_2_ in real-world devices [25]. In the calculations, the SiO_2_ region planarizes the structure. Its thickness is equal to the etching depth of RW, which is set to 660 nm throughout this paper. This etching depth corresponds to etching through all layers, from the top down to the p-Al_0.05_GaN. The etching ends 240 nm above the waveguide layer. Detailed structural information is provided in Table 1.

## 3. Numerical Model

The optical phenomena in the laser are simulated numerically with the use of the two-dimensional Plane Wave Admittance Method (PWAM) [31] where the vectorial Maxwell’s equations are solved by an algorithm based on plane wave expansion in the epitaxial plain combined with the admittance method in the vertical direction. PWAM calculates the complex effective refractive indices of the modes and corresponding distributions of the optical modes. For all calculations, the same number of plane waves was used, set to 150. This value was determined based on initial calculations performed for the SRW structure, for which the optical window was of the maximal size of one related to any structure modification considered in the analysis. Since PWAM lacks a mathematically rigorous convergence criterion, the condition used in the analysis is based on the minimum number of plane waves beyond which no changes are observed in the fifth significant digit of the real part of the effective refractive index. For the boundary conditions, absorbing perfectly matched layers (PMLs) [32] were employed. In the horizontal direction, the calculation area is 20 µm wider than an aperture, with 10 µm added to both the left and right sides. In the vertical direction, the calculation area spans from the bottom of the n-AlGaN cladding to the top electrode.

Table 1 shows the material parameters used in the optical calculations. Details on the analytical formulae for the dispersion of the refractive index in nitride materials can be found in [30]. The free-carrier absorption value is assumed and can be considered as a simple linear relation between the doping concentration and absorption [33]. In the calculations, an *x*-dependent piecewise-uniform gain distribution is assumed in the layers corresponding to the quantum wells. A positive constant gain $g_{QW}$ is applied beneath the ridge A, while a negative gain representing absorption $\alpha_{QW}$ is applied outside this region (see Figure 1). In a self-consistent simulation, where thermal, electrical, and recombination phenomena were taken into account, the situation would be different. Carrier diffusion could smooth the gain and absorption profiles, leading to some gain extending beyond the ridge A aperture and increased absorption outside that region, rather than the uniform values assumed here.

## 4. Results and Discussion

Simulations were performed for laser structures designed for blue light emission (λ=440nm), based on the devices described in [24]. The layer materials and thicknesses of the modelled structures are listed in Table 1. Ridge widths, as well as the gain $g_{QW}$ and absorption $\alpha_{QW}$ values in the active layer, are provided in the captions for all figures in this section. The coordinate system used to present the light intensity distributions is shown in Figure 1.

### 4.1. Single-Ridge-Waveguide Laser

**Figure 2:**
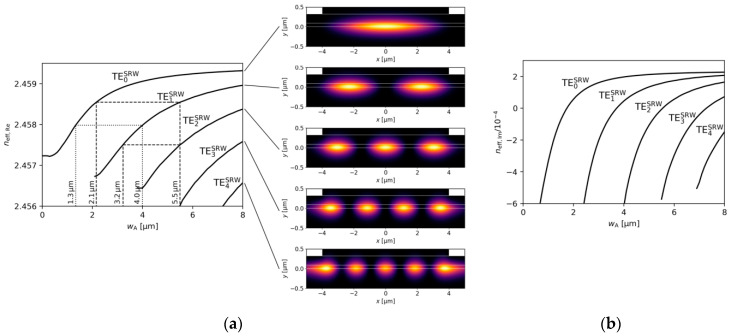
The (**a**) real and (**b**) imaginary parts of the effective refractive indices versus width of the ridge ($w_{A}$) in the SRW structure, calculated for $g_{QW}=3000\;{\mathrm{cm}}^{-1}$ and $\alpha_{QW}=10,000\;{\mathrm{cm}}^{-1}$. In (**a**), the light intensity distribution of modes in the SRW structure with $w_{A}=8\;$ µm are also illustrated.

The key parameter that determines the number of lateral modes in an SRW structure is the ridge width. The real and imaginary parts of the effective refractive index $n_{eff}$, shown as functions of the ridge width, are presented in Figure 2a and Figure 2b, respectively. Zoomed-in views, presenting the light intensity distribution for a structure with a ridge width of 8.0 µm—the largest ridge width considered here—are included as insets.

The imaginary part of the effective refractive index neff,Im is proportional to the modal gain $g_{m}$, according to the formula

(1) gm=4πλneff,Im

Its positive value indicates that for a given mode all possible optical losses induced by internal absorption and edge losses are balanced by the material gain present in the active region. The same condition indicates that a laser reaches a lasing threshold. In the case of multiple transversal modes existing in the laser for a given width of the ridge, the modification of the material gain enables a different number of modes to reach the lasing threshold. In real-world configurations, material gain is a function of the current density. Lasing is a highly nonlinear behaviour in which spatial and spectral hole burning is the most dominant effect dictating the number of modes present in the spectrum. Nevertheless, the difference in the imaginary part of the modes can be considered as a factor indicating the relative difference in current required to enable lasing of the modes. Consequently, maximizing the difference in the imaginary part of the effective index, neff,Im,0, of the fundamental mode ${TE}_{0}\;$ with respect to the mode with the next highest neff,Im,i contributes to single-mode operation, which can take place at higher injected current densities. In this paper, i is a positive integer for higher-order modes and is 0 for the fundamental mode.

The complex values for mode dispersions presented in Figure 2 reveal typical waveguide-like behaviour [34], with higher mode numbers present in the structure as the ridge width increases and lower optical losses. Figure 2 also shows that truly single-mode operation is possible for $w_{A}$ < 2.2 µm, which corresponds to a cut-off of ${\mathrm{TE}}_{1}^{\mathrm{SRW}}$ mode. However, for an assumed gain in the active region of $3000\;{cm}^{-1}$ this mode has high enough gain to be present in the lasing spectrum for $w_{A}>3.8\;µm$. For 2.2 µm< $w_{A}<3.8\;µm$ ${\mathrm{TE}}_{1}^{\mathrm{SRW}}$ mode can be present in the lasing spectrum for higher levels of gain in the active region.

To describe the discrimination of higher-order modes, a quantity called mode separation, $m_{s}$, is introduced and defined as the difference between the imaginary parts of the effective refractive index of the fundamental mode and the mode with the next highest imaginary part. This quantity is expressed as follows:

(2) ms=neff,Im,0− maxneff,Im,i

**Figure 3:**
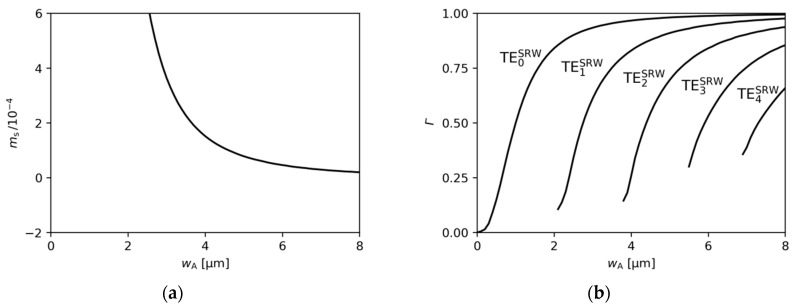
(**a**) Mode separation and (**b**) ridge A confinement factor calculated for SRW structures with various ridge A widths, obtained for $g_{QW}=3000\;{\mathrm{cm}}^{-1}$ and $\alpha_{QW}=10,000\;{\mathrm{cm}}^{-1}$.

Although the discrimination of higher-order modes will be of utmost importance for the DRW and TRW structures, the mode separation value for the SRW structure will serve as the reference value in the sections that follow. Therefore, its values for different ridge widths are presented in Figure 3a.

The primary factor determining the difference in the imaginary part of the effective refractive index between modes is the variation in the amount of the optical field penetrating into the highly absorbing regions located in the active layer outside the ridge area, where interband absorption occurs. Therefore, in the analysis of mode discrimination, it is useful to introduce a parameter that quantifies the amount of the optical field present within the gain region. The ridge A confinement factor, Γ, is introduced and defined as the portion of the light intensity confined below ridge A:

(3) $$\Gamma =\frac{\int_{x_{A,1}}^{x_{A,2}}I\left(x\right)dx}{\int_{x_{s,1}}^{x_{s,2}}I\left(x\right)dx},$$

where $x_{A,1}$ and $x_{A,2}$ correspond to the left and right boundaries of ridge A ($x_{A,2}-x_{A,1}=w_{A}$), respectively, $x_{s,1}$ and $x_{s,2}$ denote the left and right boundaries of the entire calculation area, and I(x) is the light intensity along x-axis for y corresponding to the centre of the active region. The values of the confinement factor calculated for SRW structures with various ridge A widths are presented in Figure 3b.

The typical approach used in SUSY-based laser design begins with selecting a ridge A width, for which the effective refractive indices of all supported modes are calculated. Then, a set of ridge widths is identified that is equal in number to one less than the number of modes supported by ridge A, such that each of these new ridge widths supports a mode with an effective index matching that of a specific higher-order mode from structure A. The goal of using the SUSY approach is to select the largest possible width of ridge A in order to maximize the output power emitted via the ${\mathrm{TE}}_{0}^{\mathrm{SRW}}$ mode. Based on these criteria and Figure 2, the optimal widths of the DRW structure used in the subsequent analysis should not differ significantly from $w_{A}=4.0\;µm$ and $w_{B}=1.3\;µm$. Following the same design principle, the dimensions of the optimal TRW configuration should be close to $w_{A}=5.5$ µm, $w_{B}=3.2$ µm, and $w_{C}=2.3$ µm.

In the following sections, the impact of gain and absorption in the active region on the possibility of mode coupling is analysed. Gain and absorption correspond to positive and negative values of the imaginary part of the refractive index, respectively. Therefore, changes in these parameters may, in principle, influence the effective refractive indices of the modes.

**Figure 4:**
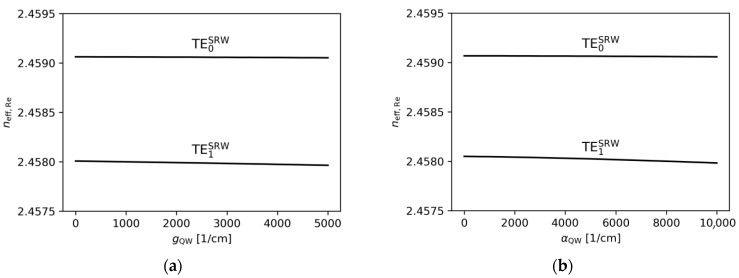
The real part of the effective refractive indices corresponding to the optical modes found for the SRW structure, obtained for $w_{A}=4.0\;µm$ and (**a**) $\alpha_{QW}=3000\;{\mathrm{cm}}^{-1}$, (**b**) $g_{QW}=10,000\;{\mathrm{cm}}^{-1}$.

**Figure 5:**
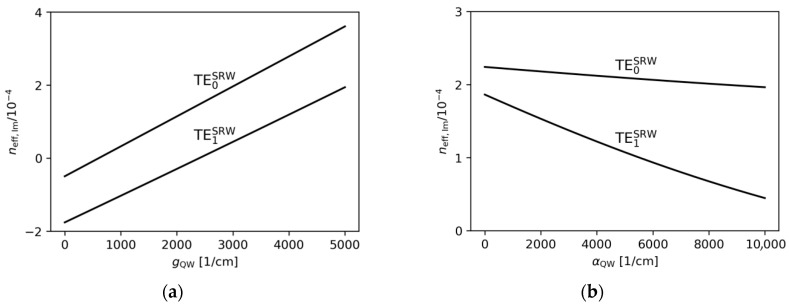
The imaginary part of the effective refractive indices corresponding to the optical modes found for the SRW structure, obtained for $w_{A}=4.0\;µm$ and (**a**) $\alpha_{QW}=3000\;{\mathrm{cm}}^{-1}$, (**b**) $g_{QW}=10,000\;{\mathrm{cm}}^{-1}$.

Figure 4a,b show the effect of varying the gain under the ridge and the absorption outside the ridge within the active region on the real part of the effective refractive index. The changes observed are very minor. This is because a variation in the gain/absorption in the range from −10^4^ cm^−1^ to 5 × 10^3^ cm^−1^ corresponds to a change in the imaginary part of the refractive index (Figure 5a,b). Therefore, from the perspective of the SUSY-based design procedure described above, changes in the imaginary part of the refractive index are assumed not to affect the selection of the optimal ridge widths.

### 4.2. Double-Ridge-Waveguide Laser

In this section, we consider the DRW structure, as well as the effective refractive indices, and mode separations. Confinement factors are analysed as the width of ridge B ($w_{B}$) varies over a broad range from 0.1 to 4.0 µm, with particular focus on three specific values: $w_{B}=0\;µm$, $w_{B}=1.4\;µm$, and $w_{B}=4.0\;µm$. Additionally, the influence of gain ($g_{QW}$) and absorption ($\alpha_{QW}$) in the active region on the analysed quantities is investigated.

**Figure 6:**
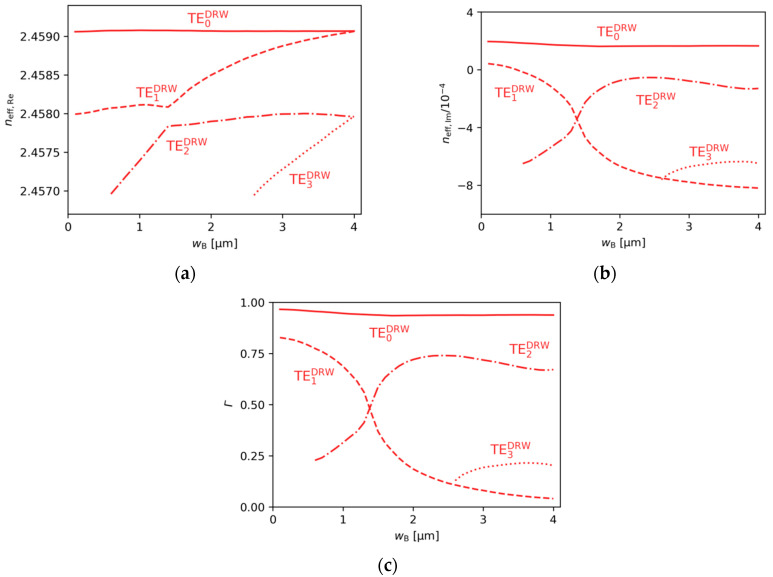
The (**a**) real and (**b**) imaginary parts of the effective refractive indices, and (**c**) ridge A confinement factor of the lateral modes present in the DRW structure, obtained for $w_{A}=4.0\;µm$, $d_{\mathrm{AB}}=0.1\;µm$, $g_{QW}=3000\;{\mathrm{cm}}^{-1}$, and $\alpha_{QW}=10,000\;{\mathrm{cm}}^{-1}$.

**Figure 7:**
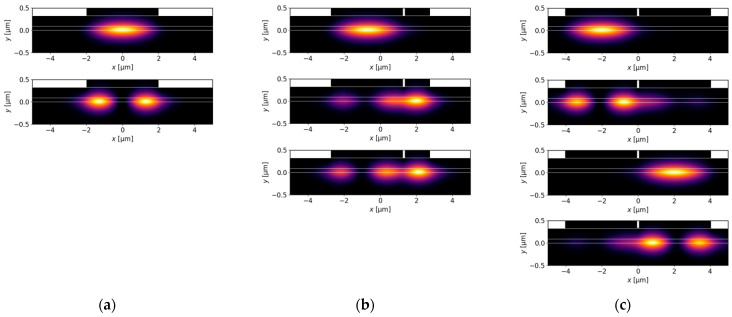
Light intensity distributions for DRW structures obtained for $w_{A}=4.0\;µm$, $d_{\mathrm{AB}}=0.1\;µm$, $g_{QW}=3000\;{\mathrm{cm}}^{-1}$, $\alpha_{QW}=10,000\;{\mathrm{cm}}^{-1}$, and various ridge B widths: (**a**) $w_{B}=0$ µm, (**b**) $w_{B}=1.4$ µm, and (**c**) $w_{B}=4.0$ µm.

Figure 6a,b show the effects of varying $w_{B}$ on the real and imaginary parts of the effective refractive index. Figure 6c presents the confinement factors Γ, which clearly indicate mode location. When $w_{B}$ approaches 0, the results for the DRW structure presented in Figure 6 become similar to those for the SRW structure with $w_{A}=4.0\;µm$ from Figure 2 and Figure 3. This is the expected result, since ridge B in the DRW structure does not significantly increase the width of the ridge region. For $w_{B}=0\;µm$, the DRW structure simplifies to the SRW structure and there are only two modes confined to the aperture of 4.0 µm (Figure 7a). The mode separation between these modes is relatively small (see Figure 2b), which may result in the excitation of higher-order modes.

At the other end of the considered $w_{B}$ range, as $w_{B}$ approaches $w_{A}$ the DRW structure consists of two ridges of similar widths, and the modes localized in ridge A and ridge B are expected to have similar effective refractive indices. Although the real parts are almost equal, the same relation is not observed for the imaginary parts. This structure is not symmetrical, because the gain is assumed below ridge A and absorption below ridge B in the active layer. Decoupled modes for $w_{B}=4.0$ µm are presented in Figure 7c, which shows that mode delocalization is not full, and a small fraction of the higher-order mode mostly located in ridge A can still be observed in the aperture related to ridge B. Consequently, in Figure 6b the imaginary part of the effective refractive index for the ${\mathrm{TE}}_{2}^{\mathrm{DRW}}$ mode does not approach that of the ${\mathrm{TE}}_{0}^{\mathrm{DRW}}$ mode at $w_{B}=4.0$ µm.

To overcome the problem of the possible excitation of higher-order modes in DRW structures, where small mode separations can be observed for narrow and wide ridge B, let us consider a ridge B with $w_{B}=1.4$ µm. According to Figure 6b, this is close to the optimal width to benefit from the SUSY effect. In the considered DRW structure, the real parts of the effective refractive indices $n_{eff,Re}$ become closer (Figure 6a) as $w_{B}$ approaches 1.4 µm, but they do not intersect due to the presence of strong coupling between the modes, which leads to anti-crossing behaviour. In contrast, both the imaginary parts of the effective indices $n_{eff,Im}$ (Figure 6b) and the confinement factors Γ (Figure 6c) can intersect. The crossings for these quantities occur because at the anti-crossing point the coupled modes acquire similar field distributions, as presented in Figure 7b, and are no longer confined to one ridge. For a ridge B with width of 1.4 µm, the effective refractive indices of modes ${\mathrm{TE}}_{1}^{\mathrm{DRW}}$ and ${\mathrm{TE}}_{2}^{\mathrm{DRW}}$ are nearly equal and the optical losses associated with the negative imaginary part of the effective refractive index (neff,Im,1, neff,Im,2) are maximized for both modes, while neff,Im,0 of fundamental mode remains positive (Figure 6b), indicating the possibility of lasing. Moreover, the ${\mathrm{TE}}_{0}^{\mathrm{DRW}}$ mode located in ridge A does not change significantly when the $w_{B}$ is modified over a broad range from 0.1 µm to 4.0 µm. The maximum relative changes in Γ for this mode, using the value calculated for $w_{B}=0.1$ µm as a reference, is only 3%. This effect enables stable single-lateral-mode operation and high emitted optical power due to the broad main ridge, which is a key feature of the SUSY effect.

In the previous section, mode separation was introduced to describe the discrimination of higher-order modes. Here, a second quantity, called the relative mode separation ($m_{\mathrm{sr}}$), is introduced, which can be used for the same purpose and is defined using the formula

(4) $$m_{\mathrm{sr}}=m_{s}/m_{s,w_{A}},$$

where $m_{s,w_{A}}$ is the mode separation calculated for the SRW structure with $w_{A}$ ridge width. Unlike mode separation, the relative mode separation provides a normalized measure, showing how the discrimination of higher-order modes in the current structure improves relative to the reference SRW structure. A value of $m_{\mathrm{sr}}$ greater than 1 indicates an enhancement in mode discrimination, while a value less than 1 indicates a reduction. This definition allows for a direct comparison of mode discrimination between different structures.

**Figure 8:**
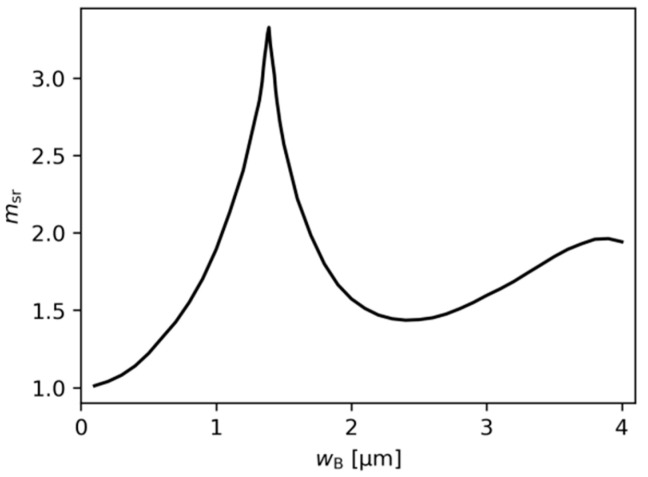
Relative mode separation calculated for DRW structures with different ridge B widths, obtained for $w_{A}=4.0\;µm$, $d_{\mathrm{AB}}=0.1\;µm$, $g_{QW}=3000\;{\mathrm{cm}}^{-1}$, and $\alpha_{QW}=10,000\;{\mathrm{cm}}^{-1}$. The mode separation calculated for the SRW structure used to find $m_{\mathrm{sr}}$ is $m_{s,4.0µm}=1.518\times 10^{-4}$ (see Figure 2b).

The relative mode separation ratio ($m_{\mathrm{sr}}$), calculated for DRW structures with ridge A width $w_{A}=4.0$ µm and various values of ridge B width ($w_{B}$), is presented in Figure 8. The maximum value of $m_{\mathrm{sr}}=3.33$ is found for $w_{B}=1.39$ µm. Figure 8 demonstrates that $m_{\mathrm{sr}}$ remains greater than 1 for all $w_{B}$ values, indicating that the discrimination of higher-order modes is stronger in the DRW configuration than in the SRW structure with the same ridge A width $w_{A}$.

**Figure 9:**
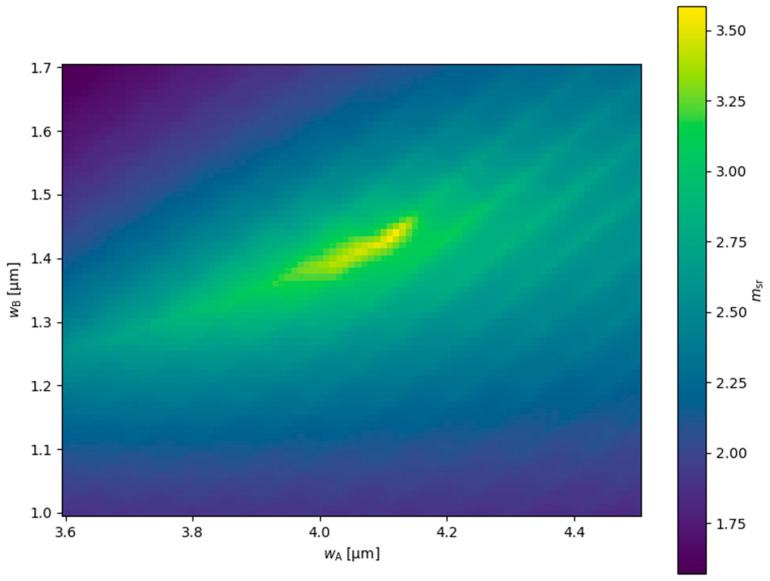
Relative mode separation calculated for DRW structures with different ridge A and ridge B widths, obtained for $d_{\mathrm{AB}}=0.1\;µm$, $g_{QW}=3000\;{\mathrm{cm}}^{-1}$, and $\alpha_{QW}=10,000\;{\mathrm{cm}}^{-1}$.

For the DRW structure, the ridge widths were further optimized to achieve higher relative mode separations. In this two-parameter analysis, ridge A widths $w_{A}$ were varied between 3.6 µm and 4.5 µm, and ridge B widths $w_{B}$ were varied between 1.0 µm and 1.7 µm. The resulting relative mode separations are shown in Figure 9. Generally, as $w_{A}$ increases the corresponding $w_{B}$ value that maximizes $m_{\mathrm{sr}}$ also increases, which is consistent with Figure 2a. For the $w_{A}$ range up to 4.12 µm, a similar trend is observed for relative mode separation. The highest $m_{\mathrm{sr}}$ value, 3.59, occurs at $w_{A}=4.12\;µm$ and $w_{B}=1.44\;µm$. For higher $w_{A}$ values, a significant decrease in $m_{\mathrm{sr}}$ is observed, which is related to the increasing $n_{eff,Im}$ of mode ${\mathrm{TE}}_{3}^{\mathrm{DRW}}$, which originates from ${\mathrm{TE}}_{2}^{\mathrm{SRW}}$ (see Figure 3).

The relative mode separation $m_{\mathrm{sr}}=3.59$ represents the highest value achievable for the DRW structure with the assumed values of $d_{\mathrm{AB}}$, $g_{QW}$, and $\alpha_{QW}$. Based on the definition of $m_{\mathrm{sr}}$ introduced in this work, this result clearly indicates that the optimized DRW structure is a more favourable choice when single-mode emission with higher output power is a priority.

Let us now analyse the effect of varying the gain and absorption parameters, which were previously assumed to be constant. Throughout this analysis, $w_{A}$ and $d_{\mathrm{AB}}$ are assumed to be the same as at the beginning of Section 4.2 ($w_{A}=4.0\;µm{,\;d}_{\mathrm{AB}}=0.1\;µm$), while $w_{B}$ is fixed at 1.4 µm.

**Figure 10:**
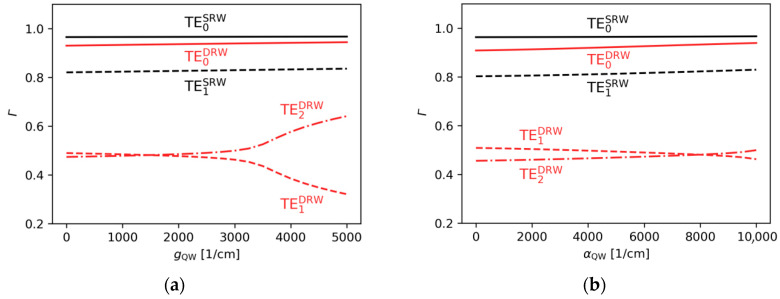
Ridge A confinement factor found for the SRW structure ($w_{A}=4.0\;µm$, black lines) and DRW structure ($w_{A}=4.0\;µm$, $d_{\mathrm{AB}}=0.1\;µm$, $w_{B}=1.4\;µm$, red lines), obtained for (**a**) $\alpha_{QW}=10,000\;{\mathrm{cm}}^{-1}$ and various gain, (**b**) $g_{QW}=3000\;{\mathrm{cm}}^{-1}$ and various absorption.

**Figure 11:**
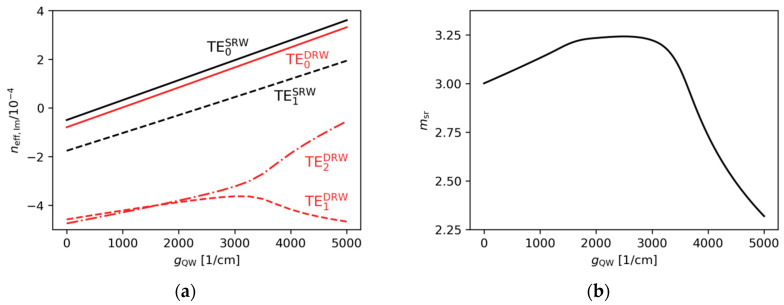
The (**a**) imaginary parts of the effective refractive indices corresponding to the optical modes found for the SRW structure ($w_{A}=4.0\;µm$, black lines) and DRW structure ($w_{A}=4.0\;µm$, $d_{\mathrm{AB}}=0.1\;µm$, $w_{B}=1.4\;µm$, red lines), obtained for $\alpha_{QW}=10,000\;{\mathrm{cm}}^{-1}$, and (**b**) relative mode separation calculated using these results.

**Figure 12:**
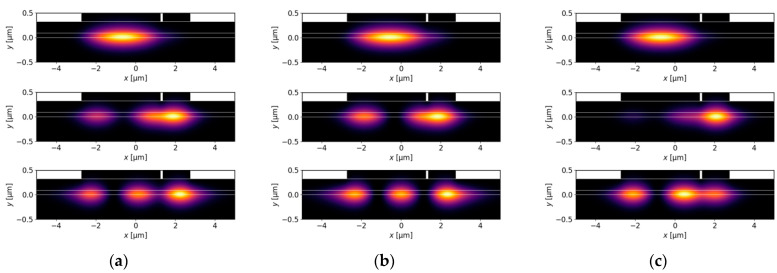
Light intensity distributions for DRW structures with $w_{A}=4.0\;µm$, $d_{\mathrm{AB}}=0.1\;µm$, and $w_{B}=1.4\;µm$, obtained for different gain and absorption values: (**a**) $g_{QW}=3000\;{\mathrm{cm}}^{-1}$ and $\alpha_{QW}=0\;{\mathrm{cm}}^{-1}$, (**b**) $g_{QW}=0\;{\mathrm{cm}}^{-1}$ and $\alpha_{QW}=10,000\;{\mathrm{cm}}^{-1}$, (**c**) $g_{QW}=5000\;{\mathrm{cm}}^{-1}$ and $\alpha_{QW}=10,000\;{\mathrm{cm}}^{-1}$.

In Figure 10a, for gain ranging from 0 to $3000\;{\mathrm{cm}}^{-1}$, more than 90% of the ${\mathrm{TE}}_{0}^{\mathrm{DRW}}$ mode is confined in the area below the ridge A, while the ${\mathrm{TE}}_{1}^{\mathrm{DRW}}$ and ${\mathrm{TE}}_{2}^{\mathrm{DRW}}$ modes are confined in less than 50%. This results in significantly lower imaginary parts of the effective refractive indices for both modes (Figure 11a). For gain of more than $3000\;{\mathrm{cm}}^{-1}$, PT symmetry breaking modifies distributions of both higher-order modes and mode discrimination is reduced. Nevertheless, in the case shown here the difference in imaginary parts of the effective refractive indices above $g_{QW}=3000\;{\mathrm{cm}}^{-1}$ for the DRW structure is larger than in the case of the SRW structure of the same width of ridge A (Figure 11a). The calculations also show that relative mode separation (Figure 11b) above $g_{QW}=3000\;{\mathrm{cm}}^{-1}$ decreases with the reduction in optical coupling between the ridges. For $g_{QW}=5000\;{\mathrm{cm}}^{-1}$ and $\alpha_{QW}=10,000\;{\mathrm{cm}}^{-1}$, the relative mode separation is only 70% of the highest relative mode separation reported here for the DRW structure with $w_{A}=4.0\;µm$, $d_{\mathrm{AB}}=0.1\;µm$, and $g_{QW}=3000\;{\mathrm{cm}}^{-1}$. This drop in the mode separation value coincides with the tendency of the mode to localize in regions corresponding to the position of ridge A or its SUSY partner ridge B (see Figure 12), which leads to a decrease in the SUSY effect.

**Figure 13:**
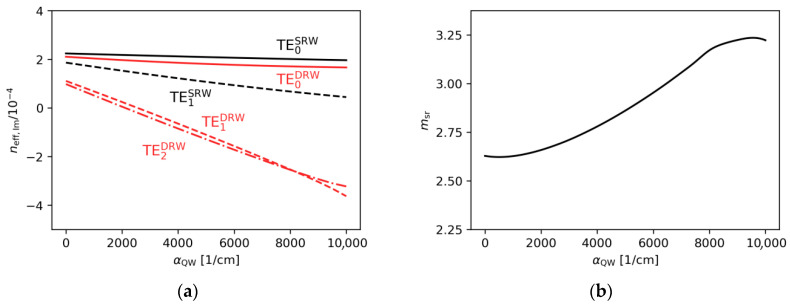
The (**a**) imaginary parts of the effective refractive indices corresponding to the optical modes found for the SRW structure ($w_{A}=4.0\;µm$, black lines) and DRW structure ($w_{A}=4.0\;µm$, $d_{\mathrm{AB}}=0.1\;µm$, $w_{B}=1.4\;µm$, red lines), obtained for $g_{QW}=3000\;{\mathrm{cm}}^{-1}$, and (**b**) relative mode separation calculated using these results.

In Figure 10b, in the absorption range from 0 to $10,000\;{\mathrm{cm}}^{-1}$, more than 90% of the ${\mathrm{TE}}_{0}^{\mathrm{DRW}}$ mode is again confined in the area below the main ridge. However, the ${\mathrm{TE}}_{1}^{\mathrm{DRW}}$ and ${\mathrm{TE}}_{2}^{\mathrm{DRW}}$ modes are always confined in less than 50%, and as a result the imaginary parts of the effective refractive indices for both modes are significantly lower for the whole considered absorption range. Again, PT symmetry breaking can be observed for this case and this effect once more modifies the distributions of both higher-order modes while reducing mode discrimination. The difference in the imaginary parts of the effective refractive indices for the DRW structure is larger than in the case of the SRW structure of the same width of ridge A (Figure 13a) for all absorptions, whereas the relative mode separation (Figure 13b) increases with absorption up to 3.25 obtained for $\alpha_{QW}=9500\;{\mathrm{cm}}^{-1}$.

### 4.3. Triple Ridge-Waveguide Laser

**Figure 14:**
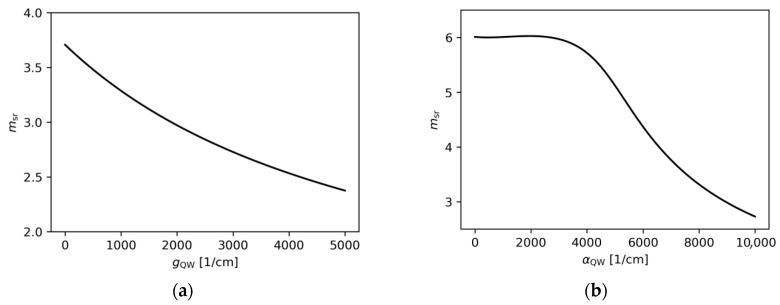
The relative mode separation calculated for TRW structures with $w_{A}=5.5\;µm$, $d_{\mathrm{AB}}=0.1\;µm$, $w_{B}=3.2\;µm$, $d_{\mathrm{AC}}=0.1\;µm$, and $w_{C}=2.3\;µm$, obtained for (**a**) $\alpha_{QW}=10,000\;{\mathrm{cm}}^{-1}$ and various gain, (**b**) $g_{QW}=3000\;{\mathrm{cm}}^{-1}$ and various absorption values. The mode separation $m_{s,5.5µm}$ calculated for the SRW structure with $w_{A}=5.5\;µm$ was used to find the relative mode separation, ($m_{\mathrm{sr}}$), in the range from $5.0\times 10^{-5}$ to $6.6\times 10^{-5}$ for (**a**), and from $1.4\times 10^{-5}$ to $6.0\times 10^{-5}$ for (**b**).

In this section, calculations are presented for the structure with three ridges. As can be seen in Figure 2a, the TRW structure can be used to discriminate two modes. When ridges B and C have optimal widths, the neff,Im of ${\mathrm{TE}}_{1}^{\mathrm{SRW}}$ and ${\mathrm{TE}}_{2}^{\mathrm{SRW}}$ modes can be significantly reduced. By ‘optimal widths’ are meant the widths $w_{B}$ and $w_{C}$ estimated in Section 4.1, after $w_{A}$ was chosen as the ridge A width. Ridges B and C are situated on opposite sides of ridge A, in order to maximize the coupling between them and ridge A. As can be seen from the results presented in Section 4.2, the optimal widths in the DRW structure do not differ much more than 0.15 µm from those derived from Figure 2a. Therefore, for the TRW structure, $w_{A}=5.5$ µm, $w_{B}=3.2$ µm, and $w_{C}=2.3$ µm are set. To reduce the number of parameters, these ridge widths are kept fixed while the impacts of gain (Figure 14a) and absorption (Figure 14b) in the active layer on the relative mode separation are investigated.

**Figure 15:**
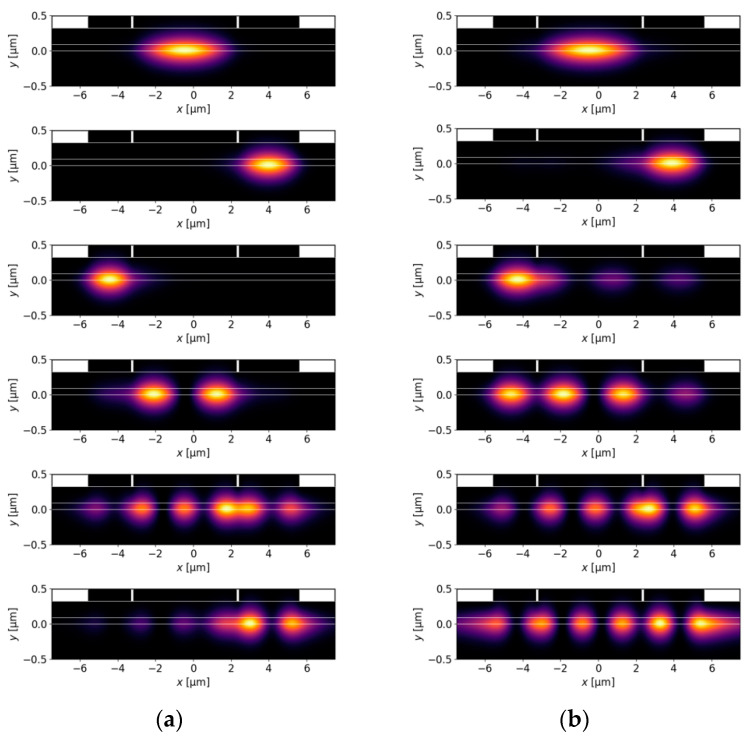
Light intensity distributions for TRW structures with $w_{A}=5.5\;µm$, $d_{\mathrm{AB}}=0.1\;µm$, $w_{B}=3.2\;µm$, $d_{\mathrm{AC}}=0.1\;µm$, and $w_{C}=2.3\;µm$, obtained for (**a**) $g_{QW}=3000\;{\mathrm{cm}}^{-1}$, $\alpha_{QW}=10,000\;{\mathrm{cm}}^{-1}$, and (**b**) $g_{QW}=3000\;{\mathrm{cm}}^{-1}$, $\alpha_{QW}=3000\;{\mathrm{cm}}^{-1}$.

To evaluate the impact of gain, the absorption value is set to the highest value considered in the previous calculations, $\alpha_{QW}=10,000\;{\mathrm{cm}}^{-1}$. In this case, as shown in Figure 14a, the highest relative mode separation is 3.71 and decreases with the increasing gain. Although $m_{sr}$ is higher than any other value for this parameter reported for the DRW structure (see Section 4.2), the relative difference is less than 4%. This limited improvement is due to the presence of a higher-order mode that is confined to the ridge A aperture. This mode, along with all other modes, is presented in Figure 15a, which demonstrates that the SUSY effect was not fully achieved.

Although Figure 13b, showing results obtained for the DRW structure, does not suggest that smaller absorption leads to higher relative mode separation, it is still worth investigating the impact of absorption values ranging from $0\;{\mathrm{cm}}^{-1}$ to $10,000\;{\mathrm{cm}}^{-1}$ on the manifestation of the SUSY effect in TRW structures. The TRW structure, in contrast to the DRW structure, exhibits distinct behaviour within this range. As shown in Figure 14b, when the assumed absorption ranges from $0\;{\mathrm{cm}}^{-1}$ up to approximately $3000\;{\mathrm{cm}}^{-1}$, the relative mode separation remains nearly constant and is close to 6.0. Figure 15b presents light intensity distributions for the TRW structure, with gain and absorption values set to $3000\;{\mathrm{cm}}^{-1}$ each. For these parameters, six confined modes are observed, which is consistent with the expected results derived from the analysis based on Figure 2a. In Figure 15b, there are two modes confined only to narrow ridges (ridge A and ridge B), three modes distributed across the whole ridge structure, and one mode confined solely to ridge A. The SUSY effect is observed, where five modes experience absorption and are consequently suppressed, leaving the fundamental mode, ${\mathrm{TE}}_{0}^{\mathrm{TRW}}$, located in ridge A, as the only emitted mode. The wider emission region is expected to enable fundamental-mode emission with high optical power.

**Figure 16:**
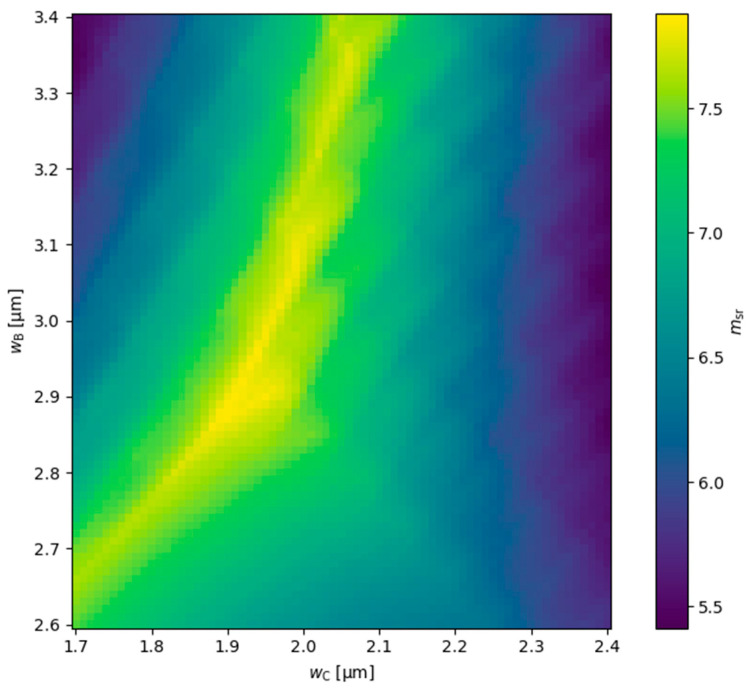
Relative mode separation calculated for TRW structures with different ridge B and ridge C widths, obtained for $w_{A}=5.5\;µm$, $d_{\mathrm{AB}}=0.1\;µm$, $d_{\mathrm{AC}}=0.1\;µm$, $g_{QW}=3000\;{\mathrm{cm}}^{-1}$, and $\alpha_{QW}=3000\;{\mathrm{cm}}^{-1}$. The mode separation calculated for the SRW structure used to find $m_{\mathrm{sr}}$ is $m_{s,5.5µm}=3.004\times 10^{-5}$.

Further improvement in the relative mode separation for the TRW structure is expected to be possible through multiparameter optimization. Figure 16 shows that, similarly to the DRW structure, the ridge widths for the TRW structure estimated based on the analysis performed for the SRW structure (Figure 2) should be considered only as a starting point for detailed calculations and are not optimal. From Figure 16, the relative mode separation can be increased to at least 7.8 for $w_{B}=2.90\;µm$ and $w_{C}=1.95\;µm$, which are slightly smaller than the initial estimates. The optimal ridge widths for the TRW structure reported here were obtained assuming a gain of $3000\;{\mathrm{cm}}^{-1}$ and absorption of $3000\;{\mathrm{cm}}^{-1}$, with the absorption value differing from that used in the SRW structure for the initial estimates. This may explain why the initial guesses were not fully accurate. A more comprehensive analysis, varying not only in terms of ridge widths but also in gain and absorption values, would provide a more precise optimization. Such a multiparameter study is significantly more time-consuming than the two-parameter calculations presented here. In this work, we aim only to highlight the potential limitations of the initial estimates.

It is also important to note that the gain and absorption values assumed in this paper are not step functions in real structures, where carrier concentrations in the active layers and resulting gain/absorption exhibit different profiles. This discrepancy may lead to smaller relative mode separations. The results presented here may be considered a starting point for investigating the SUSY effect in nitride-based structures.

## 5. Conclusions

This work presents a comprehensive multiparameter theoretical analysis of two nitride-based edge-emitting laser (EEL) configurations: the double-ridge waveguide (DRW) and the triple-ridge waveguide (TRW). Both structures were investigated with the objective of enhancing fundamental-mode emission and ensuring stable single-mode operation in devices intended for high-optical-power applications.

The study of ridge-width tolerances demonstrated that the suppression of higher-order modes strongly depends on precise geometrical design, which was achieved by fitting the ridge widths B and C. As can be seen from the mode separation ratio results, even small variations of just a few 0.1 µm in ridge width can significantly reduce the mode separation ratio, which reaches up to 50% in the case of the DRW structure. This indicates that, even though both designs can maintain robust single-mode operation, their actual performance is sensitive to fabrication accuracy.

The impact of gain and absorption in the active region was also considered. By varying their values in the active layers, the analysis revealed how gain in the active region and absorption beyond it influence mode discrimination. In this work, a step-function profile was assumed for gain and absorption, which provided a reasonable approximation of their actual distribution. The goal of this part of the study was not only to verify whether the SUSY effect can be observed for gain and absorption values within a sensible range for the considered active region, but also to identify the potential limits of these parameters beyond which the SUSY effect may be suppressed.

When comparing the two waveguide geometries, both DRW and TRW configurations were found to be capable of achieving strong mode discrimination. However, the TRW exhibited a mode separation ratio more than twice that of the DRW, underlining its potential. This makes the TRW structure particularly attractive for high-power applications where beam quality is critical. Importantly, the higher mode separation ratio of the TRW not only improves performance at nominal conditions but also increases tolerance to fabrication errors and operational instabilities, thereby enhancing device reliability.

This study also provides a framework for evaluating mode behaviour in multi-ridge nitride lasers. Even in the context of fixed-temperature calculations, there remains substantial scope for further research, as ridge widths are only one of several geometrical parameters affecting laser performance. For example, the distance between ridges, etching depth, and other structural features can be systematically varied to study their influence on mode behaviour. Moreover, it is possible to consider active regions designed for emission at different wavelengths, which opens a broad field for simulations due to the wavelength-dependent variations in refractive indices and absorption coefficients. The theoretical framework established here thus provides a foundation for future investigations into the application of SUSY concepts in nitride-based lasers.

In conclusion, this study has shown that both DRW and TRW nitride-based EELs are capable of achieving stable single-mode operation, with the TRW design offering particularly promising performance. The detailed investigation of geometrical tolerances and material effects provides practical insights for laser design, and the results reinforce the potential of nitride-based lasers as competitive high-power light sources for a wide range of advanced applications.

## Figures and Tables

**Table 1:** The composition, thickness, and room-temperature (RT) real part of refractive index and absorption coefficient of the successive layers in the GaN-based ridge-waveguide edge-emitting laser under consideration. Abbreviations: EBL—electron blocking layer; QW—quantum well; QB—quantum barrier.

Layer No.	Laser Element	Material	Thickness [nm]	Real Part of Refractive Index	Absorption Coefficient [cm^−1^]
1	Metal contact	Au	—	1.43 ^a^	5 × 10^5 a^
2	Insulation layer	SiO_2_	660	1.47 ^b^	0 ^c^
3	Contact layer	p-GaN	210	2.46 ^d^	6 ^e^
4	Cladding	p-Al_0.05_GaN	550	2.44 ^d^	6 ^e^
5	Graded cladding	p-Al_0.05_GaN → p-GaN	100	2.44 ^d^ → 2.46 ^d^	6 ^e^ → 3 ^e^
6	Superlattice EBL	p-Al_0.12_GaN	10 × 2.0 +	2.41 ^d^	40 ^e^
		p-GaN	9 × 2.0 = 38	2.46 ^d^	40 ^e^
7	Spacer	GaN	2.0	2.46 ^d^	0 ^f^
8	Waveguide	In_0.03_GaN	65	2.50 ^d^	0 ^f^
9	QB	GaN	4.0	2.46 ^d^	0 ^f^
10	QW (deep)	In_0.09_GaN	2.1	2.65 ^d^	gain
11	QW (shallow)	In_0.06_GaN	1.5	2.56 ^d^	gain
12	QB	GaN	5.0	2.46 ^d^	0 ^f^
13	QW (deep)	In_0.09_GaN	2.1	2.65 ^d^	gain
14	QW (shallow)	In_0.06_GaN	1.5	2.56 ^d^	gain
15	QB	GaN	5.0	2.46 ^d^	0 ^f^
16	Waveguide	n-In_0.03_GaN	50	2.50 ^d^	2 ^e^
17	Spacer	n-GaN	10	2.46 ^d^	4 ^e^
18	Graded cladding	n-Al_0→0.075_GaN	350	2.46 ^d^ → 2.43 ^d^	4 ^e^
19	Cladding	n-Al_0→0.075_GaN	800	2.43 ^d^	4 ^e^
20	Cladding	n-Al_0→0.025_GaN	2000	2.45 ^d^	4 ^e^

^a^ Calculated real part of refractive indices; Source: [26]; ^b^ Source: [27]; ^c^ Source: [28]; ^d^ Sources: [29] and [30]; ^e^ Assumed absorption coefficients; ^f^ Absorption coefficient is set to 0 for the undoped layers.

## Data Availability

The original contributions presented in this study are included in the article. Further inquiries can be directed to the corresponding author.

## Abbreviations

The following abbreviations are used in this manuscript:

EEL	Edge-emitting laser
SUSY	Supersymmetry
PT	Parity-time
QW	Quantum well
QB	Quantum barrier
EBL	Electron blocking layer
WG	Waveguide
SRW	Single-ridge waveguide
DRW	Double-ridge waveguide
TRW	Triple-ridge waveguide
PWAM	Plane wave admittance method
PMLs	Perfectly matched layers
RT	Room temperature
